# Synthesis and Characterization of Dual-Sensitive Fluorescent Nanogels for Enhancing Drug Delivery and Tracking Intracellular Drug Delivery

**DOI:** 10.3390/ijms18051090

**Published:** 2017-05-19

**Authors:** Szu-Yuan Wu, Tilahun Ayane Debele, Yu-Chih Kao, Hsieh-Chih Tsai

**Affiliations:** 1 Institute of Toxicology, College of Medicine, National Taiwan University, Taipei 106, Taiwan; szuyuanwu5399@gmail.com; 2 Department of Radiation Oncology, Wan Fang Hospital, Taipei Medical University, Taipei 116, Taiwan; 3 Department of Internal Medicine, School of Medicine, College of Medicine, Taipei Medical University, Taipei 110, Taiwan; 4 Department of Biotechnology, Hungkuang University, Taichung 433, Taiwan; 5 Graduate Institute of Applied Science and Technology, National Taiwan University of Science and Technology, Taipei 106, Taiwan; tilish4ayane@yahoo.com (T.A.D.); sse2a@hotmail.com (Y.-C.K.)

**Keywords:** redox-sensitive, nanogel, alginate, polyethyleneimine, fluorescent organic nanoparticles

## Abstract

Here, dual-sensitive fluorescent branched alginate-polyethyleneimine copolymer (bAPSC) nanogels were synthesized from thiolated alginate and stearoyl-derivatized branched polyethyleneimine. The formation of bAPSC conjugates was confirmed through proton nuclear magnetic resonance and Fourier transform infrared spectroscopy, whereas dynamic light scattering was used to measure the particle size and ζ potential of the nanogels. The fluorescent properties of the nanogels were confirmed through fluorescent spectroscopy and microscopy. In addition to the excitation-dependent fluorescence behavior, the fluorescence emission intensity of bAPSC was altered by both pH and γ-irradiation. This intensity was higher at a lower pH than at a higher pH, and it slightly decreased after γ-irradiation. The drug loading and encapsulation efficiency of bAPSC were 25.9% and 11.2**%,** respectively. An in vitro drug release study revealed that the synthesized nanogels release their doxorubicin (Dox) contents in a time-dependent manner, and the drug release was higher after 96 h of incubation. Approximately 43.74% and 88.36% of Dox was released after 96 h of incubation at pH 5.5 in the absence and presence of glutathione (GSH), respectively. However, relatively lower drug release, approximately 21.6% and 16%, was observed in the presence and absence of GSH at pH 7.4, respectively. Fluorescence microscopy confirmed that Dox-loaded bAPSC nanogels were internalized by HeLa cells, and drug distribution was easily tracked using fluorescent materials without additional probing agents. Moreover, cellular cytotoxicity and hemolysis results revealed less cytotoxicity and hemocompatibility of the synthesized nanogels, confirming that they are the most favorable alternative drug carriers for drug delivery systems.

## 1. Introduction

Nanotechnology has introduced several novel avenues in biological research and clinical practice [1]. Numerous nanoparticles (NPs) that have novel structural, optical, magnetic, and electronic properties relative to their bulk material counterparts have been synthesized and used in medicine as delivery vehicles, imaging probes, and scaffolds [2,3]. Although numerous polymers are available, only a few currently qualify for drug delivery in clinical practice [4,5]. Polysaccharides, highly explored natural polymers, are used to deliver one or more pharmaceutical agents, such as paclitaxel, geldanamycin, doxorubicin (Dox), and platinum derivatives for cancer treatment [6]. Once NPs are enhanced with drug-carrying and transport capabilities, imaging markers, such as fluorescent dyes, are crucial for tracking drug distribution either in vitro or in vivo [1]. Several fluorophores, such as fluorescent dyes and proteins as well as bioluminescent proteins, are conventionally used in combination with nanocarriers to track drug delivery [7]. However, fluorescent or bioluminescent proteins are highly susceptible to enzymatic degradation, lack photostability, and have an inefficient transfection process [8]. Alternative fluorescent bioprobes are quantum dots and lanthanide-doped NPs, which have high luminescence intensity [9]. However, in addition to being cytotoxic, heavy metal ions are susceptible to aggregation in cellular environments, which markedly limits their applications [10]. Therefore, over the last few decades, the development of luminescent polymeric NPs for biomedical applications has gained considerable interest because of their advantages, including excellent and tunable optical properties, surface chemistry-modifying ability, noncytotoxicity, and biodegradability.

Polyethyleneimine (PEI), a water-soluble polymer, is a highly charged polyelectrolyte with protonated nitrogen on every third atom along its backbone [11]. Branched PEI contains highly reactive tertiary, secondary, and primary amino groups, which can be protonated in different pH environments depending on their p*K*a value [12,13,14]. Because of the protonable nitrogen molecules in its backbone, PEI exhibits the proton sponge effect and absorbs protons in low-pH environments, which chronologically enhance the proton influx and cause nanogel swelling due to charge repulsion, which further disrupts the nanogels to release their cargo into the cytoplasm [15,16,17]. Furthermore, because PEI contains amine-rich nanoclusters and undergoes electron–hole recombination, it is fluorescent and can be used as an imaging probe [18]. Therefore, PEI remains the most favorable polymeric transfection agent and is extremely appealing for developing gene carriers [19,20]. However, because of its multiple amino functional groups and cationic nature, PEI, particularly branched PEI, is highly toxic and must be further derivatized with biocompatible polymers to minimize its cytotoxicity [21,22]. Polysaccharide-derivative based NPs have gained attention as a vehicle for different pharmaceutical agents because of their multiple unique functional groups and physicochemical properties, including biocompatibility and biodegradability [23,24]. Hence, the toxicity of branched PEI can be minimized through derivatization with polysaccharides, which contain multiple functional groups such as carboxyl, hydroxyl, and amine groups [14,25]. Alginate is an established biocompatible, biodegradable, and linear anion polyelectrolyte polysaccharide comprising β-d-mannuronic acid (M units) and α-l-guluronic acid (G units). Alginate has polyhydroxyl and carboxyl functional groups along its backbone, which offer several derivatives through simple modification by using either chemical or enzymatic methods [26,27]. Hence, the modification of PEI with alginate can reduce the cytotoxicity of branched cationic PEI. 

In a drug delivery system, in addition to the biocompatibility and biodegradability of the nanocarriers, the ability to encapsulate therapeutic agents is important for enhancing drug accumulation at the site of interest (i.e., tumor tissues and cancer cells) through enhanced permeability and retention (EPR) effects. Hence, in this study, stearoyl chloride was used to modify cationic PEI to increase the hydrophobicity of the NP matrix, which consequently enhances the capacity to load hydrophobic drugs.

Different strategies have been used to improve the stability of and drug release from polymeric NPs. Among these, stimulus-responsive (e.g., pH or glutathione (GSH)) cleavable cross-linking of NPs has received increasing attention in maintaining stability and preventing premature drug release, while increasing drug accumulation and release at the site of interest. The pH of normal tissues is approximately 7.4, whereas that of tumor tissues is as low as 6.0 because of hypoxia and high lactate metabolism in the tumor microenvironment. In addition, the intracellular GSH level of cancer cells is much higher than that of normal cells. Hence, the synthesis of dual-responsive NPs is crucial for enhancing the therapeutic efficacy of cancer cells.

Herein, dual-sensitive (pH and GSH) fluorescence branched alginate-polyethyleneimine copolymer (bAPSC) nanogels were synthesized from cost-effective branched PEI and alginate by using simple protocols to enhance drug delivery and simultaneously track drug distribution without using additional bioprobes. The synthesized bAPSC conjugates were characterized through Fourier transform infrared (FTIR) spectroscopy and proton nuclear magnetic resonance (^1^H-NMR). The particle size and ζ potentials were measured through dynamic light scattering (DLS), whereas the surface morphology was investigated through field-emission scanning electron microscopy (FE-SEM) and transmission electron microscopy (TEM). The fluorescence properties of bAPSC NPs were investigated through fluorescent spectroscopy and microscopy. Furthermore, drug loading (DL), drug release, cytotoxicity, and cellular uptake were investigated to evaluate the biomedical applications of the synthesized bAPSC nanogels.

## 2. Result and Discussion 

### 2.1. Characterization of bAPSC Nanogels

**Scheme 1:**
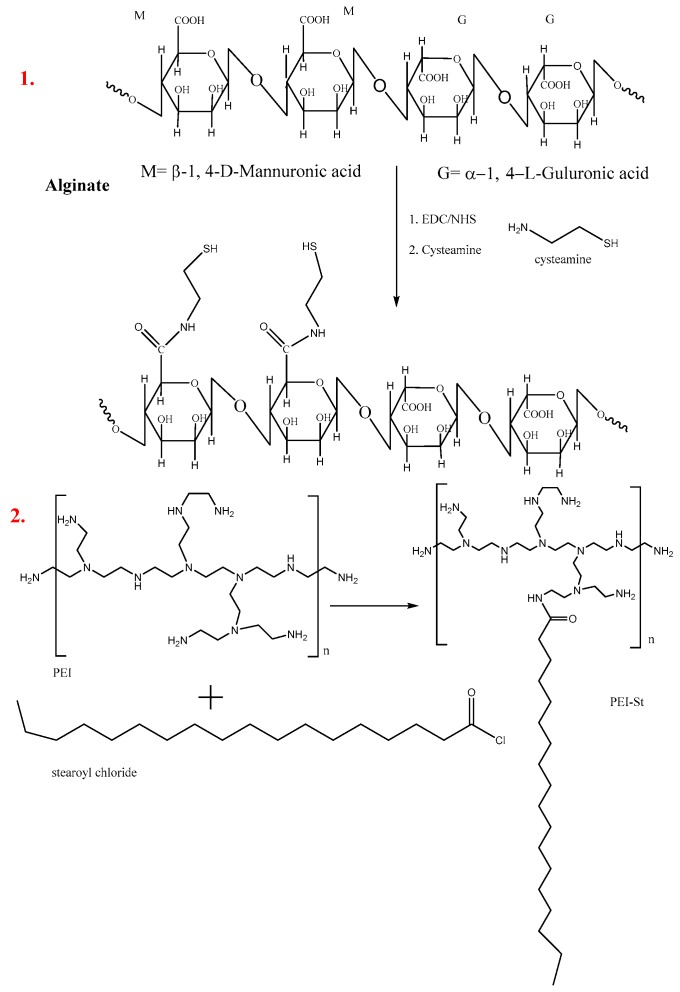

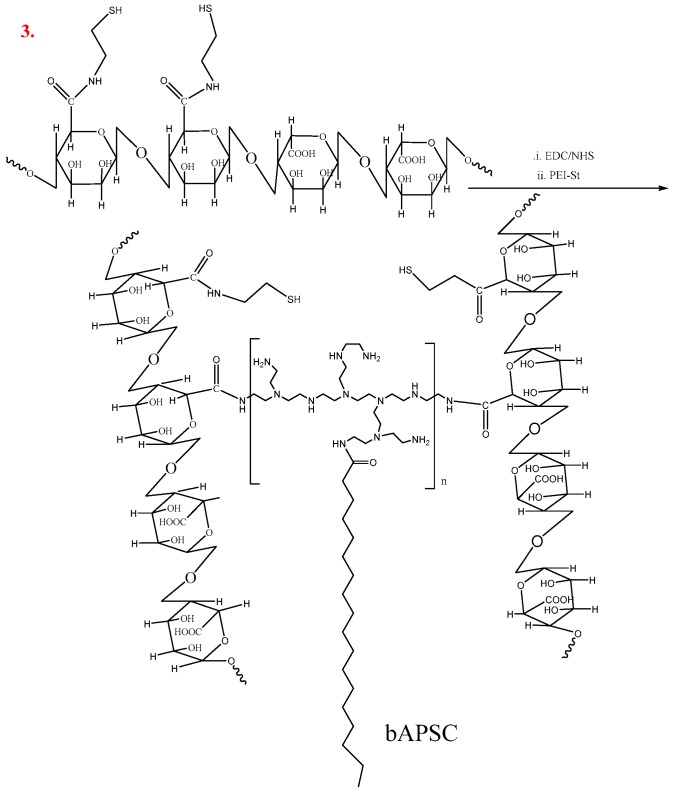
Scheme of the synthesis of dual-sensitive branched alginate-polyethyleneimine copolymer (bAPSC) conjugates. EDC: *N*-(3-dimethylaminopropyl)-*N*′-ethylcarbodiimide; NHS: *N*-hydroxysuccinimide; PEI: polyethyleneimine. 1. Synthesis of thiolated alginate; 2. Synthesis of stearoyl-derivatized PEI 3. Synthesis of bAPSC conjugates.

**Figure 1:**
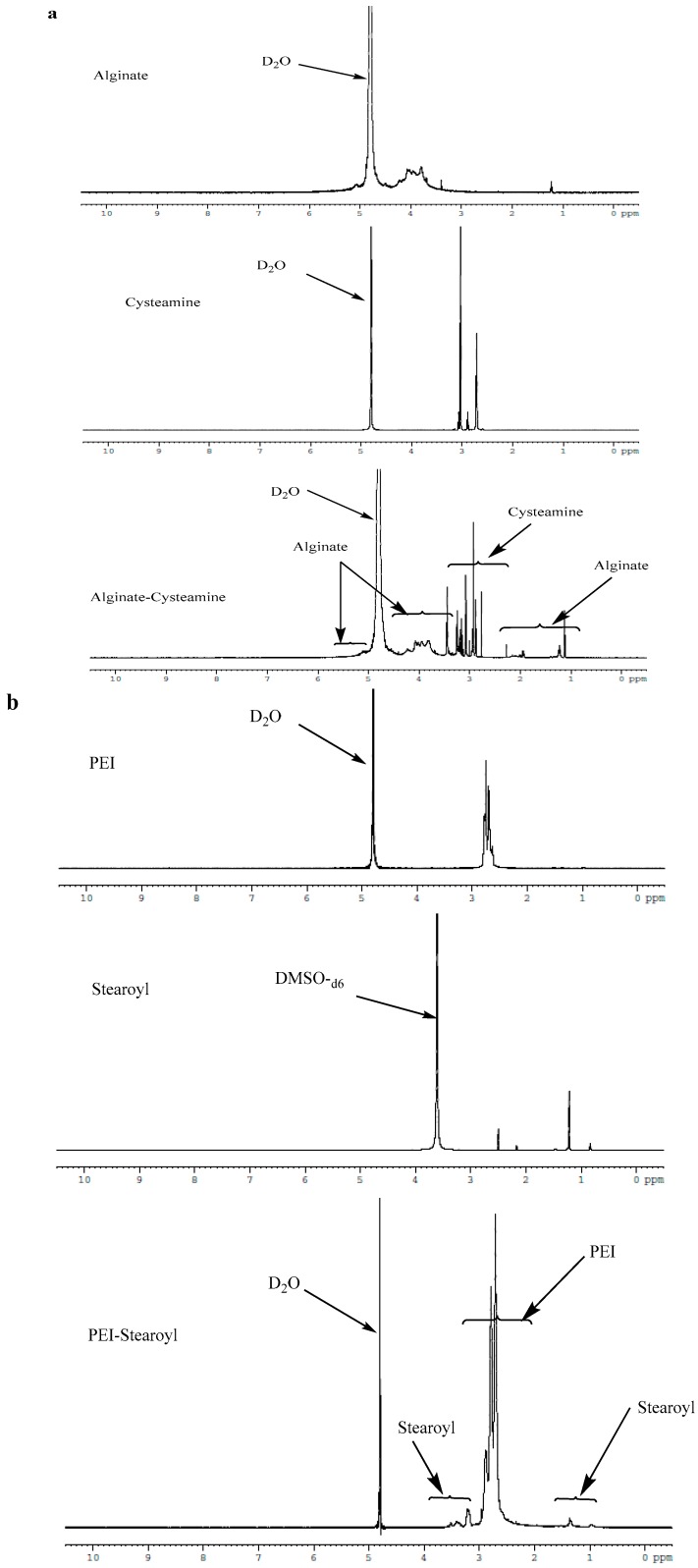

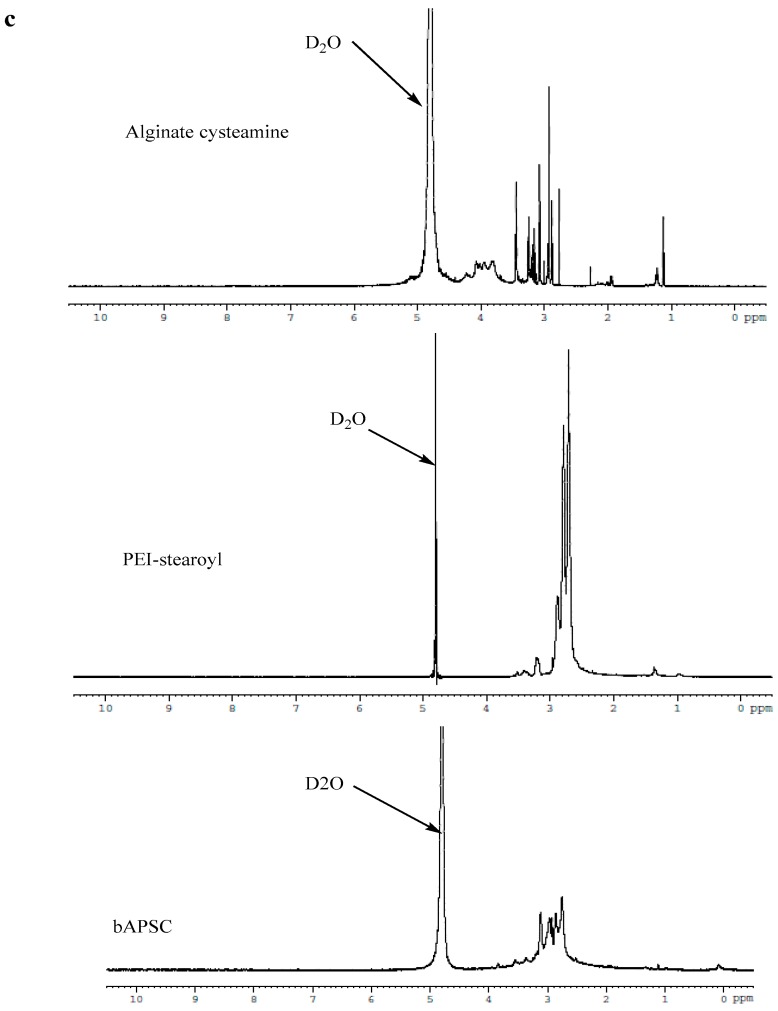
Proton nuclear magnetic resonance (^1^H-NMR) spectra of (**a**) thiolated alginate and its precursors; (**b**) derivatized polyethyleneimine (PEI) and its precursors; (**c**) bAPSC and its precursors. DMSO: dimethyl sulfoxide.

**Figure 2:**
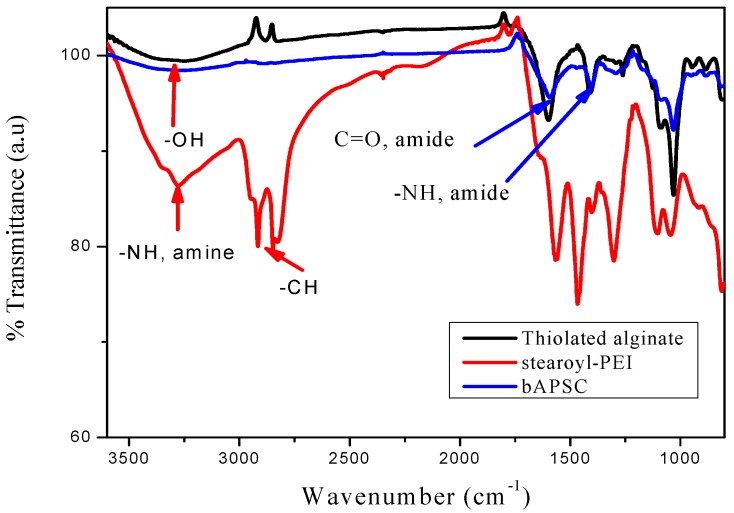
Fourier transform infrared spectroscopy (FTIR) spectra of thiolated alginate, stearoyl-PEI, and bAPSC conjugates.

The synthesis of fluorescent NPs with miscellaneous structures from PEI derivatives is widely explored in the field of nanomedicine, particularly for their use in drug delivery as carriers of different pharmaceutical agents because of the high transfection efficiency of PEI [28,29,30]. In this study, dual-sensitive bAPSC nanogels were synthesized from thiolated alginate and stearoyl-derivatized PEI through an amidation reaction by using *N*-(3-dimethylaminopropyl)-*N*′-ethylcarbodiimide (EDC)/*N*-hydroxysuccinimide (NHS). As shown in Scheme 1, bAPSC conjugates were synthesized in three simple steps. In the first step, an activated alginate carboxyl group was covalently coupled to the amine group of cysteamine via an amide linkage to form thiolated alginate by using EDC/NHS. In the second step, hydrophobically-modified PEI (25 kDa) was synthesized through the N-acylation of PEI with stearoyl chloride. In the third step, the thiolated alginate was conjugated to hydrophobically modified branched PEI via an amide linkage by using EDC and NHS to form bAPSC conjugates. The synthesis of conjugates was confirmed through ^1^H-NMR (Figure 1) and FTIR (Figure 2) spectroscopy.

In Figure 1a, the ^1^H-NMR peaks at 5.05 and 4.46 ppm can be attributed to H-l and H-5 of the G residues of alginate, respectively, whereas the peaks centered at 4.57 ppm originated from H-5 of the G residues adjacent to the M residues and from H-l of the M residues [31]. The ^1^H-NMR peaks of cysteamine were identified at 2.7–3.08 ppm in thiolated alginate, confirming the conjugation of cysteamine with alginate. Similarly, Figure 1b shows the conjugation of stearoyl with PEI. The ^1^H-NMR peaks of stearoyl were observed at 2.8, 1.54, 1.26, and 0.8 ppm in derivatized PEI, whereas those of PEI were at 2.4–3.05 ppm, confirming the conjugation of stearoyl with PEI. Moreover, the ^1^H-NMR spectra in Figure 1c clearly show the formation of a bAPSC complex. However, the proton intensity of alginate was substantially diminished, mainly because of the high molecular weight of branched PEI; this result is in concordance with those of our previous study [14].

Similarly, the FTIR spectrum (Figure 2) clearly shows the covalent conjugation of thiolated alginate with stearoyl-PEI via amide linkages. As shown in Figure 2, the FTIR peaks at 1590 and 1416 cm^−1^ show carbonyl (amide, C=O stretching) and amide (N-H stretching) groups, respectively, thus confirming bAPSC formation.

**Figure 3:**
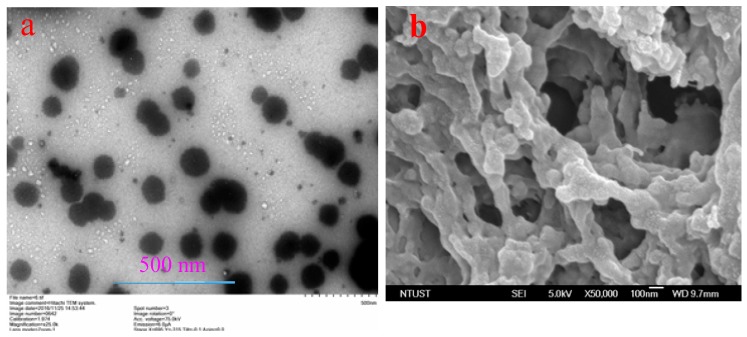
(**a**) Transmission electron microscopy (TEM) image of Dox-loaded bAPSC nanogels and (**b**) scanning electron microscopy (SEM) image of bAPSC nanogels. Dox: doxorubicin

Compared with normal tissues, tumor tissues have numerous unique characteristics, such as low extracellular pH, low oxygen tension or hypoxia, a defective vascular architecture, extensive extravasation (vascular permeability), and impaired lymphatic clearance [6]. Hence, NPs are more permeable in tumor tissues with wide endothelial gaps than in normal tissues because of EPR effects. Therefore, particle size measurement is a primary parameter in drug delivery systems, and here, the particle size and ζ potential of Dox-loaded bAPSC nanogels were measured through DLS. The average hydrodynamic diameter and ζ a potential of these nanogels were 132.3 ± 2.4 nm and −36.02 ± 2.99 mV, respectively. Moreover, TEM revealed that that the average particle size of the nanogels was <100 nm (Figure 3a). The particle size was expected to decrease because the dry-state diameter of Dox-loaded bAPSC nanogels measured through TEM was comparable with their hydrodynamic diameter determined through DLS. Moreover, as shown in Figure 3a, the TEM image of the synthesized porous nanogels (from scanning electron microscopy (SEM) images, Figure 3b) revealed a spherical morphology.

### 2.2. In Vitro Cytotoxicity of bAPSC and Its Precursors

**Figure 4:**
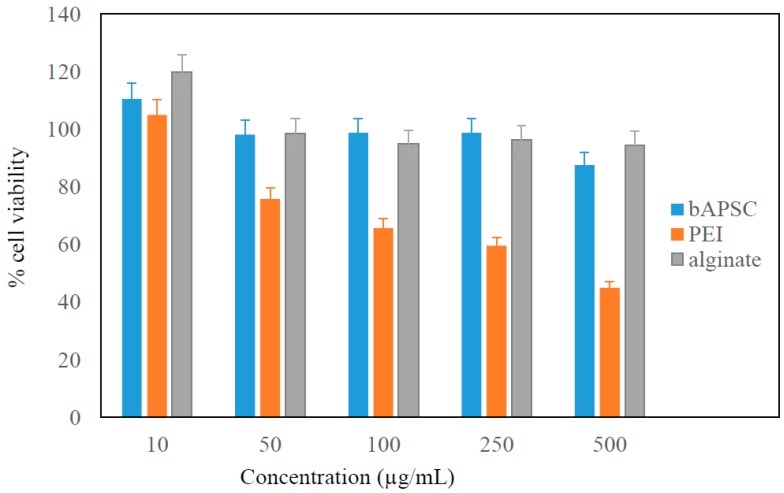
In vitro toxicity of alginate, PEI, and bAPSC in HeLa cells after 24 h of incubation. Data are presented as the mean ± standard deviation (SD) (*n* = 3).

Although PEI is widely used alone or in combination with other polymers, its toxicity is a problem because of its cationic nature [14]. To investigate this cytotoxicity, cell viability was determined after treating the cells with different concentrations of bAPSC, alginate, and PEI (Figure 4). The results confirmed that alginate is a highly biocompatible polymer, with almost >95% of cell viability being maintained in the analyzed concentration range. As expected, the cytotoxicity of PEI increased with its concentration, which might be related to its cationic amino groups. However, the cytotoxicity was decreased once PEI was conjugated with biocompatible alginate polysaccharide. The results revealed that >85% of cell viability was maintained at high concentrations of bAPSC NPs (0.5 mg/mL), whereas cell viability was nearly 45% at this point for PEI. Hence, the cytotoxicity results confirmed that the synthesized bAPSC NPs are highly biocompatible and can be used in other applicability tests.

### 2.3. In Vitro Cytotoxicity of Free Dox and Dox-Loaded bAPSC Nanogels

**Figure 5:**
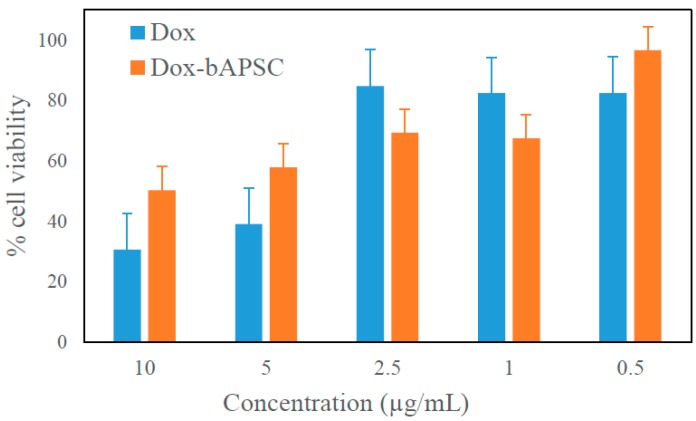
In vitro toxicity assay of HeLa cells incubated with free doxorubicin (Dox) and Dox-loaded bAPSC nanogels for 24 h. Data are expressed as the mean ± SD (*n* = 3).

The cytotoxicity of free Dox and Dox-loaded bAPSC nanogels was investigated using WST-1 (Water Soluble Tetrazolium Salts) assays by incubating HeLa cells with different concentrations of Dox for 24 h (Figure 5). The results revealed that at the same concentration, Dox-loaded bAPSC nanogels had lower cytotoxicity than did free Dox. This is because Dox is a small molecule that, when not encapsulated in nanogels, can easily permeate the cell membrane through passive diffusion and rapidly affect the cell growth.

### 2.4. Fluorescence Properties of bAPSC NPs

**Figure 6:**
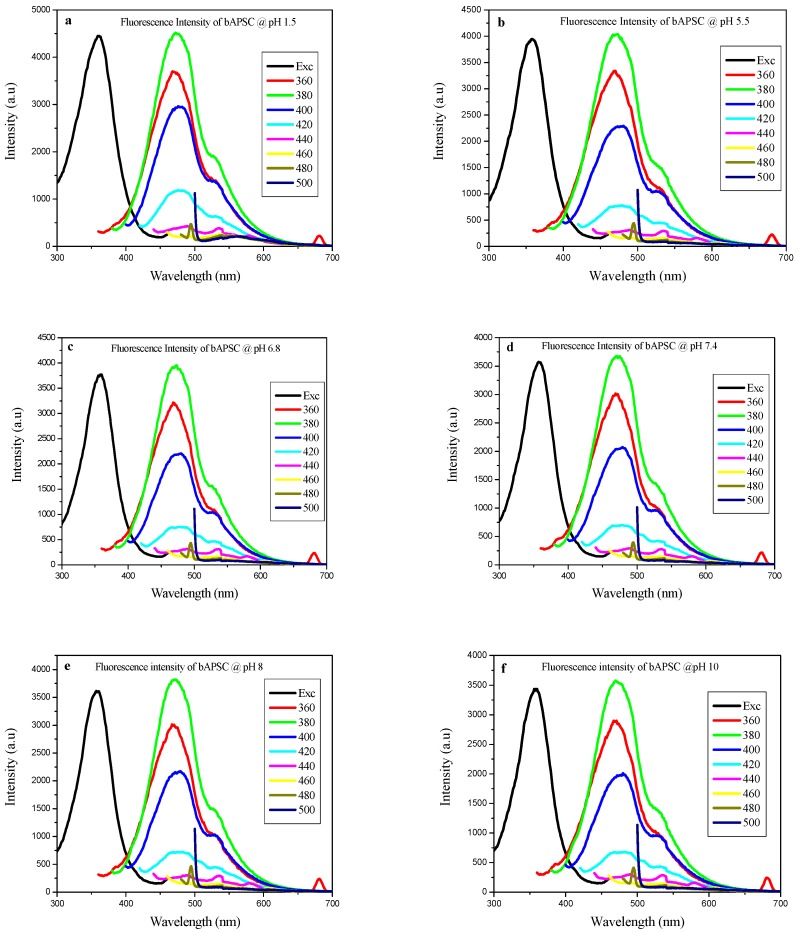
Fluorescence emission intensity of bAPSC at various pH values (**a**–**f**). The maximum excitation peak was located at 360 nm when the emission peak was set at 480 nm. When bAPSC was excited at different wavelengths (360–500 nm), the emission peaks red shifted from 480 to 550 nm.

In this study, the fluorescence properties of bAPSC NPs at different pH values were investigated in detail through fluorescence spectroscopy. As shown in Figure 6, the emission intensity (480 nm) of bAPSC NPs was the highest at the excitation wavelength of 360 nm at all tested pH values; the excitation wavelength ranged from 340 to 500 nm. With an increase in the excitation wavelength from 340 to 500 nm, the fluorescence emission intensity decreased, and the emission peak was redshifted from 480 to 550 nm, covering the wavelength range from blue to green [32,33]. In addition, similarly to the excitation-dependent fluorescence properties of carbon quantum dots, polymer dots, and other fluorescent NPs, those of bAPSC NPs were observed to be consistent with their emission spectra [33,34,35]. Although the exact mechanism remains unclear, different organic fluorescent NPs have shown similar behavior [30,32,33,36,37]. Sun et al. reported that polymer dots based on a PEI–polylactic acid (PLA) copolymer showed excitation-dependent fluorescence behavior with a significant redshift [30]. This result revealed that the rigid and compact structure of PEI–PLA NPs plays a critical role in the redshift of fluorescence emission.

**Figure 7:**
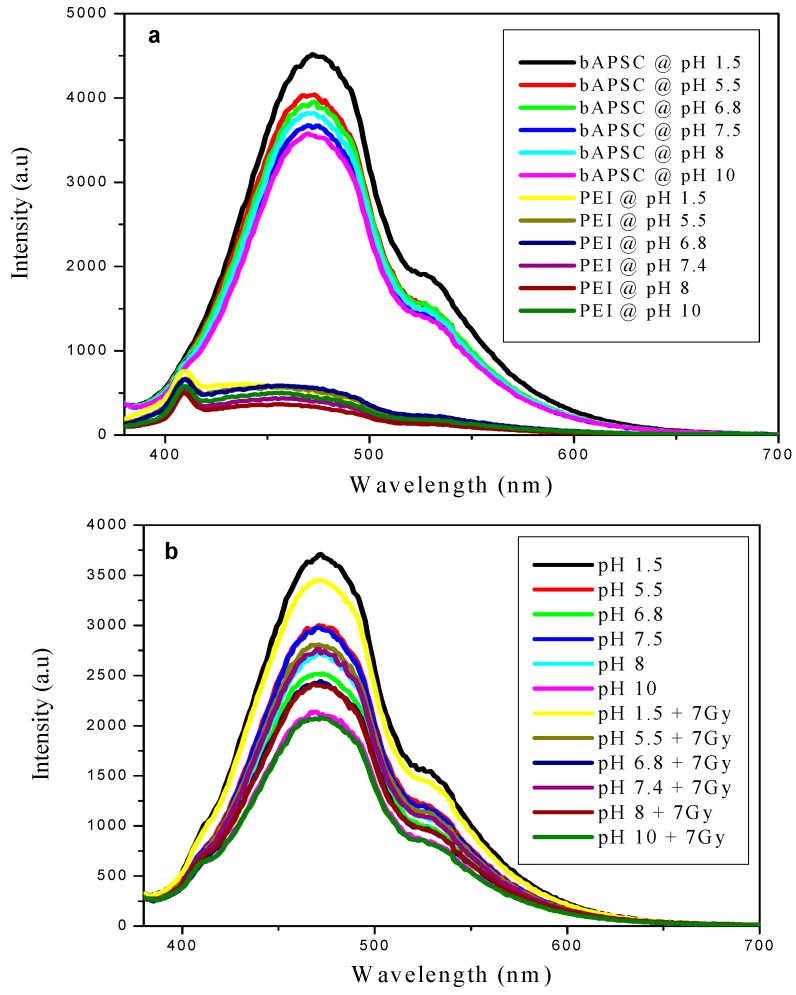
(**a**) Fluorescence emission intensity of bAPSC and PEI at various pH values; (**b**) Fluorescence emission intensity of bAPSC at various pH values in the presence and absence of γ-irradiation.

In addition to the excitation-dependent fluorescence behavior, we observed that the fluorescence intensity of bAPSC was highly sensitive to pH. As shown in Figure 7a, the fluorescence emission intensity of bAPSC was higher at a lower pH than at a higher pH, in the order pH 1.5 > 5.5 > 6.8 > 7.4 > 8 > 10. Researchers have reported that the effects of pH are related to the p*K*a value. Wang et al. investigated the effects of pH on the fluorescence emission intensity of polyamidoamine (PAMAM) dendrimers, whose structure is analogous to that of branched PEI [38]. They observed that the conformation of PAMAM was pH-responsive, and exposure to an acidic environment resulted in a more rigid and compact conformation. Hence, by considering the p*K*a values of branched PEI (4.5, 6.7, and 11.6 for primary, secondary, and tertiary amine groups, respectively) [39], it is obvious that at pH < 5, PEI exists in its fully protonated forms, and more compact NPs might be formed because of charge–charge repulsions, which may consequently enhance the fluorescence emission intensity. Moreover, we observed that compared with synthesized bAPSC NPs, branched PEI has weak fluorescence emission intensity. Lourdes et al. indicated that the fluorescence of PEI was attributed to amine-rich nanoclusters and electron–hole recombination [18].

In addition to the effects of pH, we investigated the effects of γ-irradiation on the fluorescence emission intensity of bAPSC (Figure 7b). The main characteristic peaks in the emission spectra of bAPSC NPs at various pH values after irradiation were the same as those before irradiation, and the emission intensity slightly decreased after γ-irradiation. We conjecture that γ-irradiation produces mobile charges, cations, and anions, in addition to several reactive oxygen species (ROS) [40]. Because of their instability, these radicals easily recombine to yield some novel compounds, which might quench the fluorescence emission of bAPSC NPs. Furthermore, we infer that ROS cause a disulfide cleavage, which cross-links bAPSC, consequently affecting NP conformation. Han et al. recently described markedly sensitive detection of γ-radiation through the fluorescence quenching mechanism by using imidazole-based sensor molecules [41,42].

### 2.5. In Vitro Cellular Uptake Study through Fluorescence Microscopy

**Figure 8:**
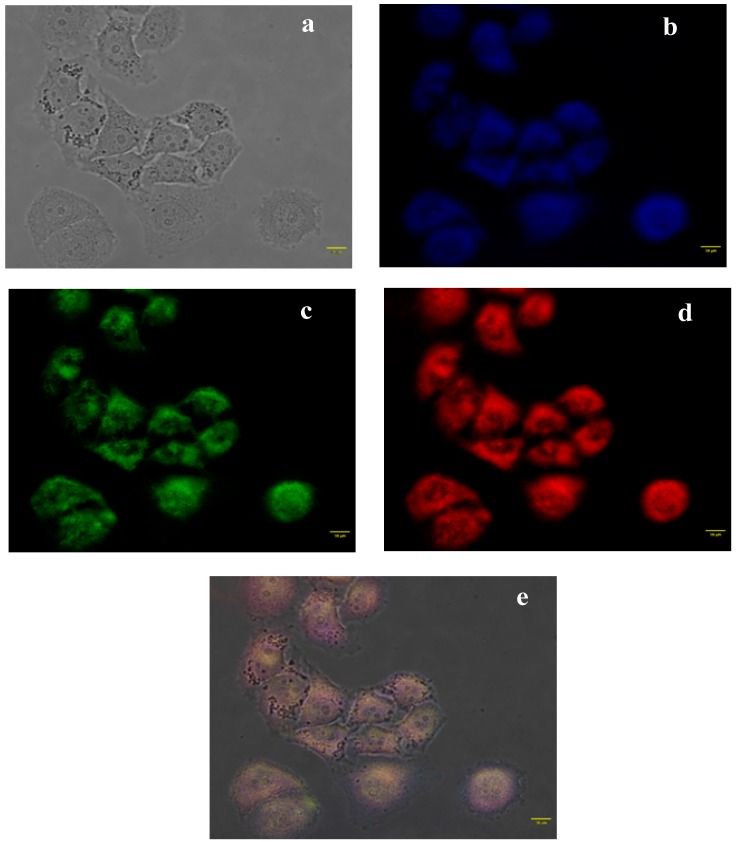
Cellular uptake of Dox-loaded bAPSC nanoparticles (NPs). (**a**) bright-field image; (**b**,**c**) blue and green fluorescence from bAPSC NPs; (**d**) red fluorescence from Dox alone; and (**e**) merged image. Scale bar = 10 μm.

Because of the unique properties of bAPSC NPs, including high water dispersibility, high fluorescence intensity (both excitation- and pH-dependent emission characteristics), and excellent biocompatibility, they were used for live cell imaging. The cellular uptake and cell imaging of bAPSC NPs was investigated through fluorescence microscopy (Figure 8). As shown in Figure 8b,c, strong blue and green fluorescence was observed in the cytoplasm of HeLa cells incubated with NPs at 500 µg/mL. Similarly, red fluorescence was observed mainly in the nucleus of the cells incubated with Dox-loaded bAPSC NPs, indicating drug release from the Dox-loaded bAPSC NPs, because Dox emits red fluorescence. Hence, based on these results, we conclude that Dox-loaded bAPSC NPs were internalized by HeLa cells, and the synthesized bAPSC NPs can be used to track drug delivery without using other probing agents. 

### 2.6. Dox Loading and Release Study

**Figure 9:**
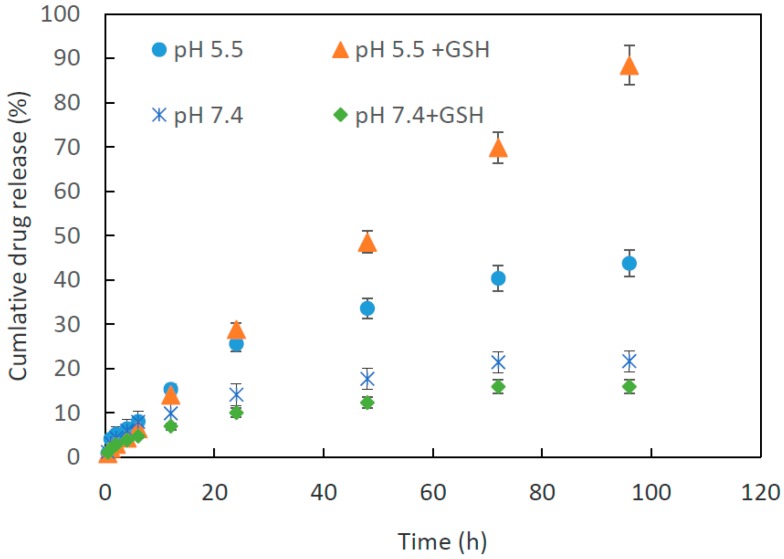
In vitro drug release from Dox-loaded bAPSC nanogels in 2-(*N*-morpholino) ethanesulfonic acid hydrate (MES) buffer (0.05 mM, pH 5.5 and 7.4) at 37 °C in the presence and absence of 5 mM glutathione (GSH).

Ultrasonication and aqueous dialysis methods were used to load Dox in the core of bAPSC NPs. The absorbance of Dox-loaded bAPSC NPs was detected through ultraviolet-visible (UV-vis) spectrophotometry. The encapsulation efficiency (EE) and Dox content of bAPSC NPs were approximately 11.2% and 25.9%, respectively, at known concentrations of Dox according to a calibration curve. Similarly, Dox release was investigated at two pH values (5.5 and 7.4) in the presence and absence of GSH. Both pH and redox potential are distinctive intracellular signals that can be exploited to activate drug release once drugs are internalized by cancer cells. Here, Dox release from dual-sensitive bAPSC nanogels was investigated by mimicking the intracellular pH and GSH levels of cancer cells. As shown in Figure 9, at pH 5.5, approximately 33.56% and 48.52% of Dox was released after 48 h in the absence and presence of GSH, respectively. Furthermore, at pH 7.4, approximately 17.69% and 12.29% of Dox was released after 48 h in the presence and absence of GSH, respectively. The synthesized nanogels released Dox in a time-dependent manner, and the release was higher after 96 h of incubation. At pH 5.5, approximately 43.74% and 88.36% of Dox was released after 96 h of incubation in the absence and presence of GSH, respectively, whereas at pH 7.4, the corresponding values were relatively lower, approximately 21.6% and 16%. Hence, this result confirmed that the synthesized bAPSC nanogels are sensitive to both pH (because of PEI and proton sponge effects) and redox potential (because of cleavage of cysteamine disulfide linkage by GSH). These results are in concordance with the prediction that reduced GSH functions act as a reducing agent in disulfide cleavage (thiol group exchange) and that branched PEI has proton sponge effects, as described in our previous report [14]. Furthermore, dual-sensitive NP synthesis is the most important factor in a drug delivery system for enhancing drug release in comparison with that of single-parameter detection modes. In summary, low pH and high GSH levels in cancer cells influence the rate of Dox release from bAPSC nanogels.

### 2.7. Cell Apoptosis/Necrosis Quantification through Flow Cytometry

**Figure 10:**
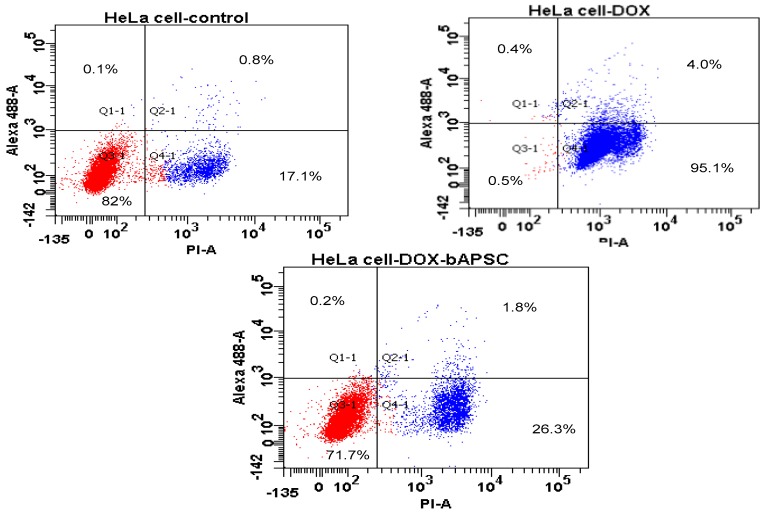
Apoptosis of HeLa cells after 6 h of incubation with media (as the control), free Dox, and Dox-loaded bAPSC nanogels, as determined through flow cytometry with Alexa Fluor 488 annexin V and propidium iodide (PI) staining. The lower and upper left quadrants in each section indicate the percentage of normal and early apoptotic cells, respectively, whereas the lower and upper right quadrants in each panel indicate the percentage of late apoptotic cells. Note: The Dox concentration was 20 µg/mL.

Flow cytometry was used to evaluate the cytotoxicity of free Dox and Dox-loaded bAPSC nanogels after double-staining HeLa cells for assessing their viability (negative for propidium iodide (PI) and weak annexin V staining), death, and apoptosis (positive for both Alexa Fluor 488 annexin V and PI). As shown in Figure 10, the cell viability varied for cells incubated for 6 h with free Dox and Dox-loaded bAPSC nanogels. The percentage of early apoptotic cells after free Dox and Dox-loaded bAPSC nanogel treatments was 0.4% and 0.2%, respectively, whereas the corresponding percentage of late apoptotic cells was approximately 99.1% and 28.1%, respectively, after 6 h of incubation. The percentage of late apoptotic cells treated with free Dox was much higher than that of cells treated with Dox-loaded bAPSC nanogels. This is because Dox is a small molecule that, when not encapsulated, can easily permeate the cell membrane through passive diffusion and instantly affect the cell growth. Furthermore, the drug release results revealed very low Dox release within 6 h of incubation, supporting the flow cytometry results (i.e., high viability of HeLa cells after 6 h of incubation with Dox-loaded bAPSC nanogels compared with free Dox). The in vitro cytotoxicity of cells treated with free Dox and Dox-loaded bAPSC nanogels was assessed after 24 h of incubation by using the WST-1 assay, as mentioned previously. For flow cytometry, we incubated the cells for only 6 h to validate the effects of incubation time. As observed for the concentration, incubation time affects cell viability.

### 2.8. Hemocompatibility Test 

**Figure 11:**
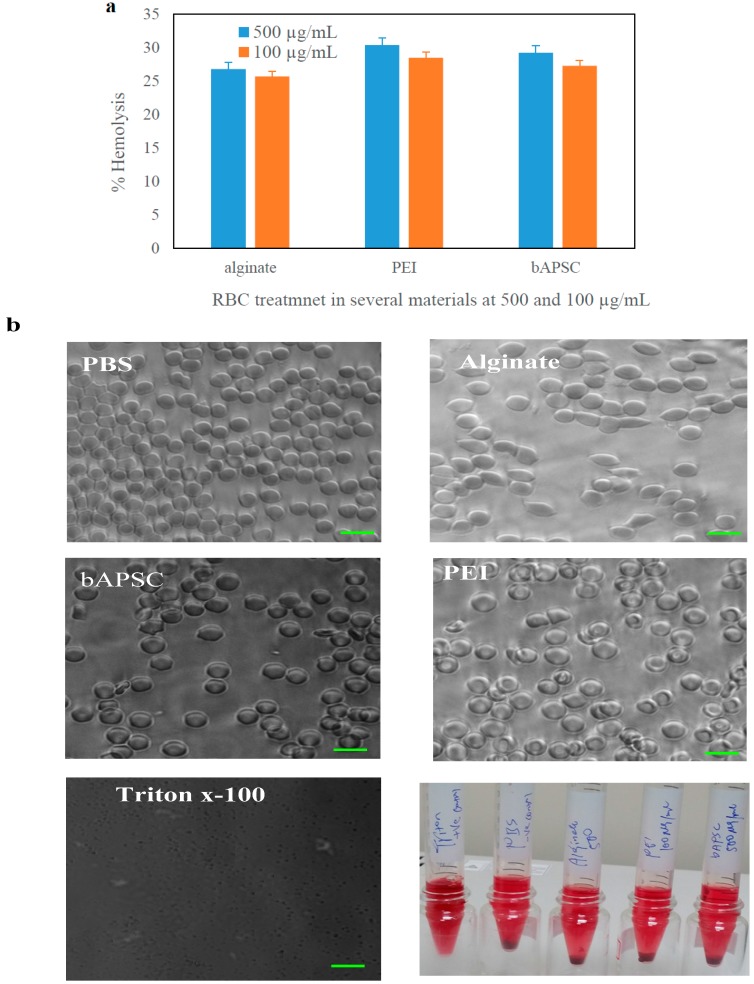
(**a**) Percentage of hemolysis and (**b**) images of erythrocytes treated with different formulations at concentrations of 500 and 100 μg/mL. Data are presented as the mean ± SD (*n* = 3) for panel a. Scale bars, 10 μm.

Hemocompatibility tests are crucial for evaluating critical interactions between foreign materials and blood cells, such as erythrocytes [43]. Here, a hemolytic assay was performed to determine the toxicity of the precursors and prepared materials against erythrocytes isolated from mouse blood. As shown in Figure 11a, the percentage of hemolysis was nearly 25% for all tested materials (i.e., at 500 and 100 μg/mL of alginate, PEI, and bAPSC). Several researchers have reported hemolysis percentages of 10% and 25% as relative cutoffs (i.e., <10% is considered nonhemolytic, whereas >25% is considered hemolytic) in the hemolysis assay, although the hemolytic cutoff varies among species (i.e., approximately 10% for humans, 10–29% for dogs, and 0–37% for rabbits) [44]. Moreover, microscopic examination and images revealed slight changes in the membrane structure of the erythrocytes after exposure to those materials (Figure 11b); the changes were similar to those in the negative control (phosphate-buffered saline (PBS), pH 7.4). However, the erythrocytes treated with Triton X-100 (positive control) showed complete cell lysis.

## 3. Materials and Methods

### 3.1. Materials

Sodium alginate (10 kDa), branched PEI (25 kDa), stearoyl chloride, cysteamine, EDC, NHS, 2-(*N*-morpholino) ethanesulfonic acid hydrate (MES), GSH, dl-dithiothreitol, Dox, and the cell proliferation reagents WST-1 and dimethyl sulfoxide (DMSO) were purchased from Sigma Chemical Co. (St. Louis, MO, USA). Dulbecco modified Eagle medium (DMEM), penicillin, sodium pyruvate, trypsin, sterilized Phosphate-buffered saline (PBS), fetal bovine serum (FBS), and l-glutamine were purchased from Gibco (USA). A dialysis bag (molecular weight (*M*_w_) cutoff: 1, 6–8, and 12–14 kDa) was purchased from CelluSept T1. The other chemical reagents and buffer solution components were analytical-grade preparations. Distilled and deionized water was used in all experiments.

### 3.2. Synthesis of Thiolated Alginate by Using Cysteamine

Alginate thiolation was achieved through amide bond formation between the activated carboxyl group of the alginate and amino group of the cysteamine by using EDC and NHS [45]. Briefly, sodium alginate (200 mg) was dissolved in 50 mL of MES buffer (0.1 M, pH 5.5) and reacted with EDC (86 mg) and NHS (64.5 mg) at room temperature for 6 h. Cysteamine was added to the aforementioned solution at an alginate-to-cysteamine ratio of 2:1; the reaction was continued for 24 h under stirring at room temperature. The resultant solutions were dialyzed for 2 days to remove byproducts and uncoupled compounds and were then lyophilized. The alginate–cysteamine conjugate was stored at 4 °C until further use. The chemical structure of thiolated alginate was confirmed through FTIR and ^1^H-NMR.

### 3.3. Synthesis of Modified PEI-Stearoyl Chloride

Hydrophobically modified PEI (25 kDa) was synthesized through the *N*-acylation of PEI with stearoyl chloride (1:20 mole/mole ratio of stearoyl chloride to ethylenimine) [46]. Briefly, 200 mg of PEI was dissolved in 10 mL of DMSO under stirring at room temperature. Similarly, stearoyl chloride (9.5 mg) was dissolved in 5 mL of DMSO under stirring at room temperature to obtain a clear solution. Subsequently, the PEI solution was slowly added to the stearoyl chloride solution under stirring at room temperature for 24 h. The resultant solutions were dialyzed for 2 days to remove byproducts and uncoupled compounds and were then lyophilized. The PEI-stearoyl chloride (PS) conjugates were stored at 4 °C until further use. The chemical structure of PS was confirmed through FTIR and ^1^H-NMR.

### 3.4. Synthesis of Redox-Sensitive Alginate-PS Conjugate

The thiolated alginate was conjugated with hydrophobically modified branched PEI via amide linkages by using EDC and NHS to yield bAPSC conjugates, as shown in Scheme 1. Briefly, 50.0 mg of thiolated alginate was dissolved in 0.1 M MES buffer, and the pH was adjusted to 5.5. EDC (21.5 mg) and NHS (10 mg) were added based on the free carboxyl group of alginate; the reaction mixture was stirred for 6 h at room temperature. Subsequently, hydrophobically-modified PS was added to the mixture under continuous stirring at room temperature for 24 h (1:0.5 *w*/*w* ratio of thiolated alginate to PS). The bAPSC conjugates were purified through dialysis for 48 h by using a dialysis membrane (*M*_w_ cutoff: 12–14 kDa) against an excess volume of distilled water. Finally, the bAPSC solution was freeze-dried (Virtis Advantage 2.0, London, UK), and the bAPSC conjugate powder was stored at 4 °C until use.

### 3.5. Characterization of bAPSC Nanogels

#### 3.5.1. FTIR Analysis

We placed 2–3 drops of alginate, PEI, cysteamine, stearoyl chloride, and bAPSC solution on the center of a salt plate (CaF_2_); the homogeneous film was stored overnight in a vacuum oven, and the sample was dried. This sample was analyzed through FTIR.

#### 3.5.2. ^1^H-NMR Analysis

The ^1^H-NMR spectra for all reaction products were recorded using Varian Unity-600 (600 MHz) NMR spectrometers (Varian, Inc., Palo Alto, CA, USA). Alginate, PEI, bAPSC, and cysteamine dissolved in D_2_O and stearoyl acid dissolved in DMSO-d6 were analyzed through NMR spectrometry.

### 3.6. Synthesis of bAPSC Nanogels

bAPSC nanogels were prepared through covalent interactions, self-assembling, and electrostatic interactions through probe sonication (in DMSO) and aqueous dialysis [47]. Briefly, 20 mg of bAPSC conjugate was dissolved in 5 mL of DMSO and 2 mL of distilled water with gentle stirring at 37 °C to obtain a clear solution. This was followed by probe sonication for 30 min. The resultant mixture was dialyzed using a dialysis tube (*M*_w_ cutoff: 6–8 kDa) against distilled water for 24 h with dH_2_O exchange at intervals of 6 h. Finally, the solution of self-assembled NPs was filtered using a syringe filter (pore size = 450 nm) to remove any impurities, and the filtrate was freeze-dried.

#### 3.6.1. Dynamic Light Scattering

The particle size and ζ potential of Dox-loaded bAPSC nanogels were measured using a Malvern Nano ZS 90 Zetasizer (Malvern, UK) operating at 25 °C.

#### 3.6.2. Field-Emission Scanning Electron Microscopy

The freeze-dried nanogel powders were placed directly on silicon wafers. The nanogel morphology was subsequently analyzed after platinum sputtering by using a JEOL JSM-6500F field-emission scanning electron microscope (JEOL, Tokyo, Japan) operating at 5.0 kV.

#### 3.6.3. Transmission Electron Microscopy

Dox-loaded bAPSC nanogels (1 mg/mL) were dispersed in dH_2_O through sonication. Subsequently, a drop of Dox-loaded bAPSC nanogels was drop-casted onto a carbon-coated copper grid, and the image was viewed after negative staining with 2% phosphotungstic acid.

### 3.7. Fluorescence Spectrophotometer 

The fluorescence intensity of bAPSC nanogels was assessed using a JASCO FP-8300 spectrophotometer (Jasco, Hachioji, Japan) equipped with a xenon lamp power supply and 1-cm path quartz cell, at Ex and Em bandwidths of 10 nm, a response of 0.1 s, medium sensitivity, a data interval of 1 nm, and a scan speed of 1000 nm/min.

### 3.8. Drug Loading

Dox was encapsulated in bAPSC NPs by using sonication and dialysis methods [48]. Briefly, 15 mg of bAPSC was solubilized in 2 mL of DMSO, and 4 mL of Dox (1 mg/mL in DMSO and neutralized with 20 μL of triethylamine) was added and vigorously stirred for 4 h at room temperature. Subsequently, 2 mL of dH_2_O was added under sonication for 3 h. For further oxidation of the thiol groups (i.e., to form disulfide linkages) of the bAPSC complex, 1 mL of H_2_O_2_ was added. The residual DMSO and unloaded Dox were eliminated through exhaustive dialysis (*M*_w_ cutoff: 6000 Da) against distilled water for 24 h, with dH_2_O exchange every 6 h. The final products were deep-frozen and collected after lyophilization. The EE and DL capacity were calculated using the following equation:

$$\mathrm{EE}\;\left(\text{\%}\right)=\frac{\mathrm{Final}\;\mathrm{fluorescence}\;\mathrm{intensity}\;\mathrm{of}\;\mathrm{Dox}\;\mathrm{in}\;\mathrm{bAPSC}\;}{\mathrm{Initial}\;\mathrm{fluorescence}\;\mathrm{intensity}\;\mathrm{of}\;\mathrm{Dox}\;\mathrm{used}}\times 100$$

$$\mathrm{DL}\;\left(\text{\%}\right)=\frac{\mathrm{Amount}\;\mathrm{of}\;\mathrm{Dox}\;}{\mathrm{Amount}\;\mathrm{of}\;\mathrm{Dox}+\mathrm{bAPSC}}\times 100$$

### 3.9. Drug Release Study

The in vitro release of Dox was assessed in 0.05 M MES buffer at pH 7.4 and 5.5 in the presence and absence of 5 mM GSH by using the dialysis method [14]. Briefly, 2 mL of the Dox-loaded bAPSC solution was collected from the DL steps after dialysis, and the amount of Dox release was monitored using a dialysis membrane (*M*_w_ cutoff: 6–8 kDa) against 10 mL of a corresponding release medium at 37 °C. After a predetermined period, the absorbance of Dox in the release medium (3 mL) was measured, and the medium was replaced with an equal amount of a fresh release medium. The Dox concentration in the sampled release medium was determined by measuring the absorbance at intervals of 450–800 nm, and the concentration was calculated using a pre-established calibration curve for Dox. The results are expressed as the percent cumulative release over time ± standard deviation for three experiments [49].

### 3.10. In Vitro Cytotoxicity of Materials, Free Dox, and Dox-Loaded bAPSC Nanogels

The cytotoxicity of the synthesized bAPSC nanogels and the precursors alginate and PEI was investigated [14]. Briefly, HeLa cells were cultured in DMEM supplemented with 10% FBS, 1% penicillin, 1% glutamine, and 1% sodium pyruvate at 37 °C and 5% CO_2_. The cells were seeded in a 96-well plate at a density of 1 × 10^4^ cells/well in a complete DMEM medium and maintained in a humidified incubator at 37 °C in 5% CO_2_ for 24 h. Serial dilutions of bAPSC, alginate, and PEI (0.01, 0.05, 0.1, 0.25, and 0.5 mg/mL) were added to the culture medium in each well and incubated at 37 °C for 24 h. After 24 h of incubation, the culture medium was replaced with a fresh medium containing 20 μL of WST-1 cell proliferation reagents and further incubated for 4 h at 37 °C and 5% CO_2_. Subsequently, the 96-well plate was shaken thoroughly for 1 min on a shaker, and the absorbance was read at test and reference wavelengths of 450 and 620 nm, respectively, by using an enzyme-linked immunosorbent assay reader (Power Wave XS, BioTek, Winooski, VT, USA). The results are expressed as the viable percentage of cells after various treatments relative to the control. The same experimental protocols were used to study the in vitro toxicity of free Dox and Dox-loaded bAPSC nanogels (Dox concentrations: 0.1, 1, 2.5, 5, and 10 µg/mL).

$$\mathrm{Cell}\;\mathrm{viability}\;\left(\text{\%}\right)=\frac{\mathrm{Absorbance}\;\mathrm{of}\;\mathrm{test}\;\mathrm{cell}}{\mathrm{Absorbance}\;\mathrm{of}\;\mathrm{control}\;\mathrm{cell}}\times 100$$

$$\mathrm{Cell}\;\mathrm{viability}\;\left(\text{\%}\right)=\frac{\mathrm{Absorbance}\;\mathrm{of}\;\mathrm{test}\;\mathrm{cell}}{\mathrm{Absorbance}\;\mathrm{of}\;\mathrm{control}\;\mathrm{cell}}\times 100$$

### 3.11. Cellular Uptake Study

HeLa cells were cultured in DMEM supplemented with 10% FBS, 1% penicillin, 1% glutamine, and 1% sodium pyruvate at 37 °C and 5% CO_2_ [14]. The cells were seeded in a 35-mm glass-bottom culture dish (MatTek Corporation, Ashland, MA, USA) at a density of 2.5 × 10^5^ cells per well and incubated at 37 °C with 5% CO_2_ for 24 h. Subsequently, the cells were co-incubated with Dox-loaded bAPSC nanogels (Dox concentration: 20 µg/mL) for 12 h at 37 °C. After the incubation, the cells were washed with PBS three times. Cellular internalization was visualized through fluorescence microscopy.

### 3.12. Cell Apoptosis/Necrosis Quantification through Flow Cytometry 

The Alexa Fluor 488 annexin V apoptosis detection kit was used to quantify apoptotic and necrotic cells by using the standard fluorescence-activated cell sorting assay [46,50]. Briefly, HeLa cells were seeded in six-well plates at a density of 1 × 10^5^ cells per well for 24 h and washed three times with PBS to remove dead cells. The cells were incubated with free Dox and Dox-loaded bAPSC nanogels (Dox concentration: 20 µg/mL) for 6 h. They were subsequently washed, collected, and resuspended in 500 μL of 1× annexin-binding buffer. Alexa Fluor 488 annexin V and PI were added according to the manufacturer’s recommendations. The samples were incubated in the dark for 15 min at room temperature. An additional 400 μL of 1× annexin-binding buffer was added and mixed gently with the samples before analysis.

### 3.13. Hemolytic Assay

To investigate the interactions of alginate, PEI, and the synthesized bAPSC NPs with blood erythrocytes, we performed hemolytic assays [46,51]. Briefly, 200 µL of alginate, PEI, and bAPSC (at 500 and 100 µg/mL) was incubated with a 1% suspension of red blood cells obtained from mice for 1 h at 37 °C. Subsequently, each sample was centrifuged at 3000 rpm for 15 min. The absorbance of the supernatant was analyzed at 540 nm through UV-vis spectrophotometry, and the precipitated erythrocytes were observed under a microscope. The hemolysis percentage was calculated according to the following formula:

$$\mathrm{Hemolysis}\;\left(\text{\%}\right)=\frac{\mathrm{Asample}}{\mathrm{Atriton}\;x\_100}\times 100$$

A suspension of red blood cells treated with Triton X-100 was used as the positive control.

## 4. Conclusions

In this study, biocompatible dual-sensitive fluorescent bAPSC nanogels with a particle size of 132.3 ± 2.4 nm and negative ζ potential of −36.02 ± 2.99 mV were synthesized. The fluorescent properties of the nanogels were confirmed through fluorescent spectroscopy, and the results revealed that the nanogels exhibit blue to green fluorescence emission behavior. Similar to other fluorescent NPs, the nanogels showed excitation-dependent fluorescence properties. In addition to the excitation-dependent fluorescence behavior, we observed that the fluorescence emission intensity of the nanogels was altered by pH and γ-irradiation. The fluorescence emission intensity was higher at a lower pH than at a higher pH and slightly decreased after γ-irradiation. The encapsulation efficiency and DL capacity of the nanogels were 11.2% and 25.9%, respectively. An in vitro drug release study revealed that the synthesized nanogels release Dox in a time-dependent manner, and the release was higher after 96 h of incubation in the presence of GSH at pH 5.5. Similarly, the fluorescence property of the nanogels was confirmed through fluorescence microscopy, which revealed that Dox-loaded bAPSC nanogels were internalized by HeLa cells. In addition, hemolytic assays confirmed the hemocompatibility of the synthesized nanogels.
